# Direct RNA Sequencing of the Coding Complete Influenza A Virus Genome

**DOI:** 10.1038/s41598-018-32615-8

**Published:** 2018-09-26

**Authors:** Matthew W. Keller, Benjamin L. Rambo-Martin, Malania M. Wilson, Callie A. Ridenour, Samuel S. Shepard, Thomas J. Stark, Elizabeth B. Neuhaus, Vivien G. Dugan, David E. Wentworth, John R. Barnes

**Affiliations:** 1 Oak Ridge Institute of Science and Education (ORISE), Oak Ridge, Tennessee USA; 2 Battelle Memorial Institute, Atlanta, Georgia USA; 3 Influenza Division, National Center for Immunization and Respiratory Diseases (NCIRD), Centers for Disease Control and Prevention (CDC), Atlanta, Georgia USA

## Abstract

For the first time, a coding complete genome of an RNA virus has been sequenced in its original form. Previously, RNA was sequenced by the chemical degradation of radiolabeled RNA, a difficult method that produced only short sequences. Instead, RNA has usually been sequenced indirectly by copying it into cDNA, which is often amplified to dsDNA by PCR and subsequently analyzed using a variety of DNA sequencing methods. We designed an adapter to short highly conserved termini of the influenza A virus genome to target the (-) sense RNA into a protein nanopore on the Oxford Nanopore MinION sequencing platform. Utilizing this method with total RNA extracted from the allantoic fluid of influenza rA/Puerto Rico/8/1934 (H1N1) virus infected chicken eggs (EID_50_ 6.8 × 10^9^), we demonstrate successful sequencing of the coding complete influenza A virus genome with 100% nucleotide coverage, 99% consensus identity, and 99% of reads mapped to influenza A virus. By utilizing the same methodology one can redesign the adapter in order to expand the targets to include viral mRNA and (+) sense cRNA, which are essential to the viral life cycle, or other pathogens. This approach also has the potential to identify and quantify splice variants and base modifications, which are not practically measurable with current methods.

## Introduction

Decades ago, a method was published describing the use of base-specific chemical degradation with chromatographic and autoradiographic resolution as a way of directly sequencing short stretches of RNA^1^. Since then, little progress has been made on directly sequencing RNA. Instead, the elucidation of RNA sequences is typically indirect and primarily requires methods that synthesize cDNA from RNA templates. While these methods are powerful^2^, they suffer from limitations inherent to cDNA synthesis and amplification such as template switching^3^, artifactual splicing^4^, loss of strandedness information^5^, obscuring of base modifications^6^, and propagation of error^7^. In 2009, a method for RNA sequencing was developed on the Helicos Genetic Analysis System where poly(A) mRNA is sequenced by the step-wise synthesis and imaging of nucleotides labeled with an interfering but cleavable fluorescent dye^8^. While the input material requirements for this method are extremely low, the long workflow and short reads are limiting. Nevertheless, these approaches expose two major limitations of RNA sequencing: sequencing by synthesis and short read length. Overall, current technologies for sequencing RNA templates present difficulties in the assessment of base modifications, splice variants, and analysis of single RNA molecules.

**Figure 1:**
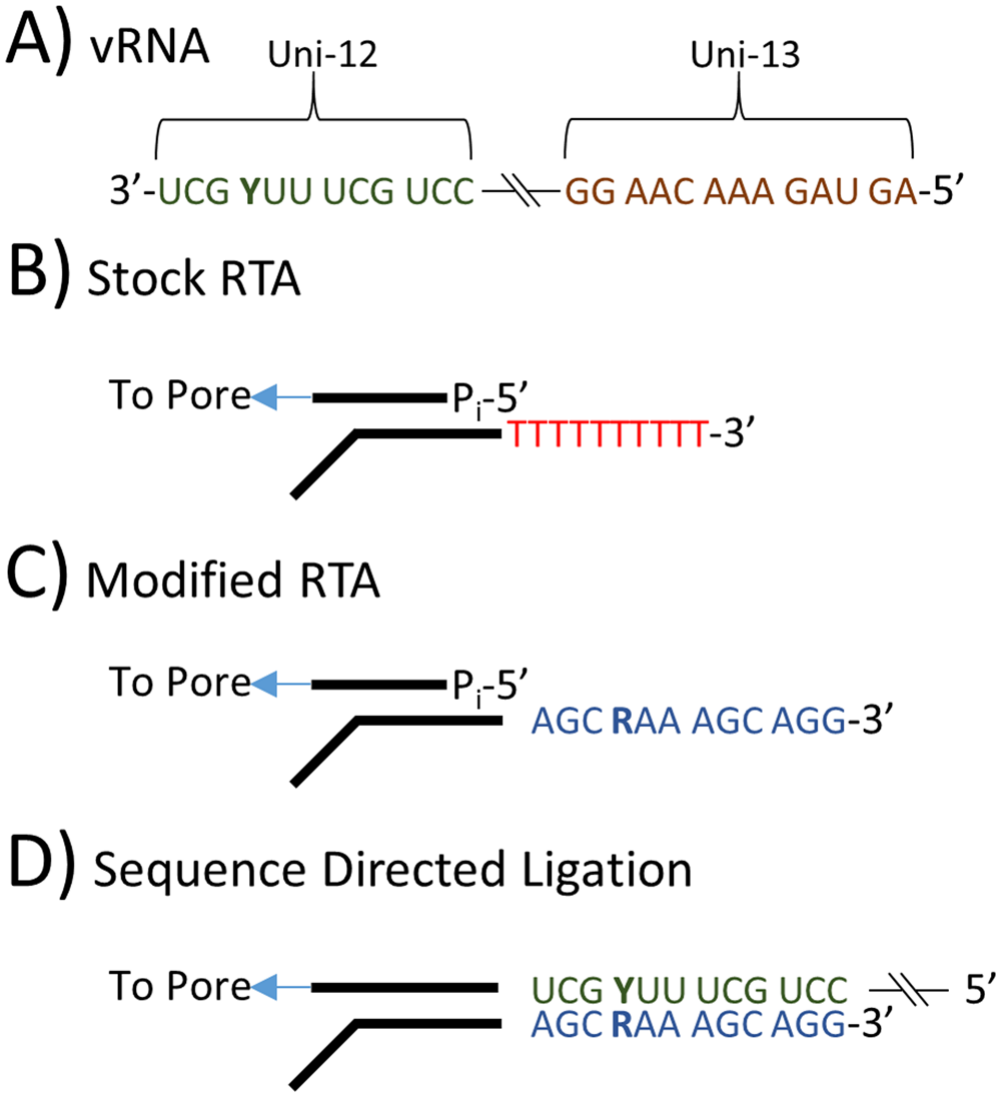
(**A**) Influenza A viruses contain highly conserved 12 and 13 nt sequences at the 3′ and 5′ termini. (**B**) The key component of Oxford Nanopore direct RNA sequencing is a Reverse Transcriptase Adapter (RTA) which targets poly(**A**) mRNA and is ligated to the 3′ end of the mRNA. A sequencing adapter is then ligated to the RTA which directs the RNA strand into the pore for sequencing. (**C**) The RTA was modified to target the 3′ conserved 12 nt of the influenza A virus genome. (**D**) The modified RTA hybridizes and is ligated to vRNA in the first step of direct RNA sequencing.

Influenza A viruses are negative-sense segmented RNA viruses^9–11^. Sequencing these viruses has played an important role in their understanding for 40 years^12,13^ including the discovery of highly conserved vRNA termini^14^ (Fig. 1A). These 3′ and 5′ termini are 12 and 13 nucleotides in length, respectively, and they are highly conserved across the PB2, PB1, PA, HA, NP, NA, M, and NS genome segments of influenza A viruses, which enabled the development of a universal primer set for influenza A virus genome amplification^15,16^. Even though these conserved vRNA termini have been readily exploited for efficient and sensitive next generation sequencing (NGS) of influenza A virus segments^16–18^, current methods retain some of the limitations inherent to cDNA-based techniques^3–7^. A new tool for long read direct RNA sequencing could reduce these biases and greatly aid efforts to directly sequence influenza A viruses and other RNA viruses.

Oxford Nanopore Technologies (ONT) recently released their direct RNA sequencing protocol. This method involves the sequential ligations of a reverse transcriptase adapter (RTA) and a sequencing adapter^19^. The RTA is a small dsDNA molecule (Fig. 1B) that contains a T_10_ overhang designed to hybridize with poly(A) mRNA and a 5′ phosphate (P_i_) that ligates to the RNA creating a DNA-RNA hybrid. The RTA also serves as a priming location for reverse transcription of the entire length of the RNA molecule, though the cDNA generated is not sequenced. The DNA-RNA hybrid is then ligated to the sequencing adapter which directs the RNA strand of the assembled library into the nanopore for sequencing^19^.

We describe direct RNA sequencing of five influenza A virus genomes through modification of recently released RNA methods from Oxford Nanopore Technologies^19^ (Fig. 1C) by targeting the conserved 3′ end of the influenza A virus genome with an adapter to capture it (Fig. 1D), rather than a primer to amplify it. The efficacy of the adapter is tested by sequencing the RNA genome of an influenza A virus generated by reverse genetics A/Puerto Rico/8/1934 (H1N1) as well as genetically diverse contemporary human or avian influenza A viruses including A/Florida/20/2018 (H1N1pdm09), A/Texas/50/2012 (H3N2), A/chicken Ghana/20/2015 (H5N1), and A/British Columbia/1/2015 (H7N9) (Table 1). The total RNA was purified from either allantoic fluid harvested from infected embryonated chicken eggs or infected MDCK cell culture supernatants. The results from the nanopore sequencing are compared to the current Illumina-based pipeline utilized by the Influenza Genomics Team at the Centers for Disease Control and Prevention.

**Table 1:** Influenza rA/Puerto Rico/8/1934 virus, a common laboratory strain and candidate vaccine virus backbone, was used in this study to demonstrate direct RNA sequencing effectiveness and repeatability using a crude starting material.

Influenza A Virus	Subtype	Volume	Titer*	Purpose in Study
rA/Puerto Rico/8/1934	H1N1	200 µL	4.2 × 10^11^	Pure Virus
rA/Puerto Rico/8/1934	H1N1	1,500 µL	6.8 × 10^9^	Crude Virus Triplicates
A/Florida/20/2018	H1N1pdm09	2,100 µL	3.2 × 10^7^	Contemporary virus & LOD
A/Texas/50/2012	H3N2	500 µL	3.5 × 10^6^	Contemporary virus
A/chicken Ghana/20/2015	HPAI H5N1	500 µL	4.3 × 10^7^	Contemporary virus
A/British Columbia/1/2015	LPAI H7N9	500 µL	3.2 × 10^8^	Contemporary virus

Influenza A/Florida/20/2018 (H1N1pdm09), A/Texas/50/2012 (H3N2), A/chicken Ghana/20/2015 (HPAI H5N1) and A/British Columbia/1/2015 (LPAI H7N9) viruses were used to demonstrate this method’s broad utility across contemporary influenza A viruses of current clinical significance. Influenza A/Florida/20/2018 (H1N1pdm09) virus was also used to determine the limit of detection.

*Influenza A/Florida/20/2018 (H1N1pdm09), A/Texas/50/2012 (H3N2), and A/British Columbia/1/2015 (LPAI H7N9) viruses were propagated in MDCK cells and the titer is presented as a TCID_50_. Influenza rA/Puerto Rico/8/1934 (H1N1) and A/chicken Ghana/20/2015 (HPAI H5N1) viruses were propagated in embryonated chicken eggs and the titers are presented as EID50_50_.

## Results

### RNA calibration strand: enolase II mRNA

First, the RNA calibration strand enolase was directly sequenced on the MinION platform. Three sequencing experiments covered 100% of the coding regions of the 1,314 nucleotide long RNA molecule to an average depth of 122,207 ± 8,126 (sd). Of the 171,135 ± 21,987 reads, 98.6 ± 1.4% mapped to the reference sequence (Tables 2 and S3), with 100% of the mapped reads in the sense orientation. The direction of the reads and the positive slope of the coverage diagram (Fig. S1) are indicative of directional sequencing of mRNA from the 3′ end. The distribution of read lengths (Table S1 and Fig. S2) accurately corresponds to the expected length of 1,314 nucleotides. The read level accuracy was 90.4 ± 0.8%, and the consensus sequence was 99.72% ± 0.04% in concordance with the known reference.

**Table 2:** An individual MiSeq experiment of influenza rA/Puerto Rico/8/1934 (H1N1) vRNA from crude virus is compared to MinION experiments of enolase mRNA (technical triplicate), influenza rA/Puerto Rico/8/1934 (H1N1) vRNA from crude virus (triplicate), and single runs of crude material containing influenza A/Florida/20/2018 (H1N1pdm09), A/Texas/50/2012 (H3N2), A/chicken Ghana/20/2015 (HPAI H5N1), and A/British Columbia/1/2015 (LPAI H7N9) viruses. Values from triplicate experiments are presented as averages ± standard deviation. This data is expanded in Table S3.

	MiSeq	MinION					
	rA/Puerto Rico/8/1934 Crude	Enolase	rA/Puerto Rico/8/1934 Crude	H1N1pdm09	H3N2	HPAI H5N1	LPAI H7N9
Reads	143,572	171,135 ± 21,987	54,353 ± 15,314	106,425	277,452	1,741	2,949
Mapped	143,378	169,041 ± 23,467	53,721 ± 15,145	103,913	277,269	1,709	2,837
% Mapped	99.9%	98.6 ± 1.4%	98.8 ± 0.1%	97.6%	99.9%	98.2%	96.2%
Accuracy	99.6%	90.4 ± 0.8%	86.2 ± 0.31%	86.0%	85.9%	85.0%	85.5%
Insertion	0.30%	1.49 ± 0.02%	1.66 ± 0.01%	2.0%	1.6%	3.8%	3.3%
Deletion	0.06%	5.4 ± 0.5%	8.2 ± 0.2%	7.6%	8.2%	7.9%	7.6%
Substitution	0.36%	4.7 ± 0.4%	6.4 ± 0.1%	7.3%	6.8%	8.1%	7.8%
Consensus	≡100%	99.72% ± 0.04%	98.97 ± 0.01%	98.28%	98.30%	97.45%	98.31%

### Sequencing RNA from crude versus purified influenza rA/Puerto Rico/8/1934 (H1N1) virus

Based on available details on the RTA system, it was possible to make further modification to target other RNA species (Fig. 1). To adapt this technique for the influenza A virus genome, the target sequence of the RTA was changed from an oligo-dT to a sequence complementary to the 12 nucleotides that are conserved at the 3′ end of the RNA segments of influenza A viruses (Table S2).

As a favorable substrate for the modified adapter and a positive control for future experiments, RNA from two sucrose purified influenza rA/Puerto Rico/8/1934 (H1N1) virus (EID_50_ 4.2 × 10^11^) preparations (pure) were sequenced via MinION. Two sequencing experiments covered 100% of the coding regions of the PB2, PB1, PA, HA, NP, NA, M, and NS vRNA segments to an average depth 8,360 and 936 respectively (Fig. S3). Of the 119,350 and 13,721 reads acquired in each run, 99.6 and 99.1% mapped to influenza rA/Puerto Rico/8/1934 (H1N1) virus, respectively (Table S3), in a roughly even distribution among the eight vRNA segments (Fig. S4) with 100% of the mapped reads in the negative-sense orientation. The distribution of read lengths (Fig. S5 and Table S1) corresponds to expected lengths of each respective segment. The read level accuracies for the two runs were 85.2 and 83.8%, and the consensus sequences were 98.7 and 98.5% in concordance with consensus sequence generated using our standardized M-RTPCR^15,16^ amplified genome and MiSeq approach (Table S3).

**Figure 2:**
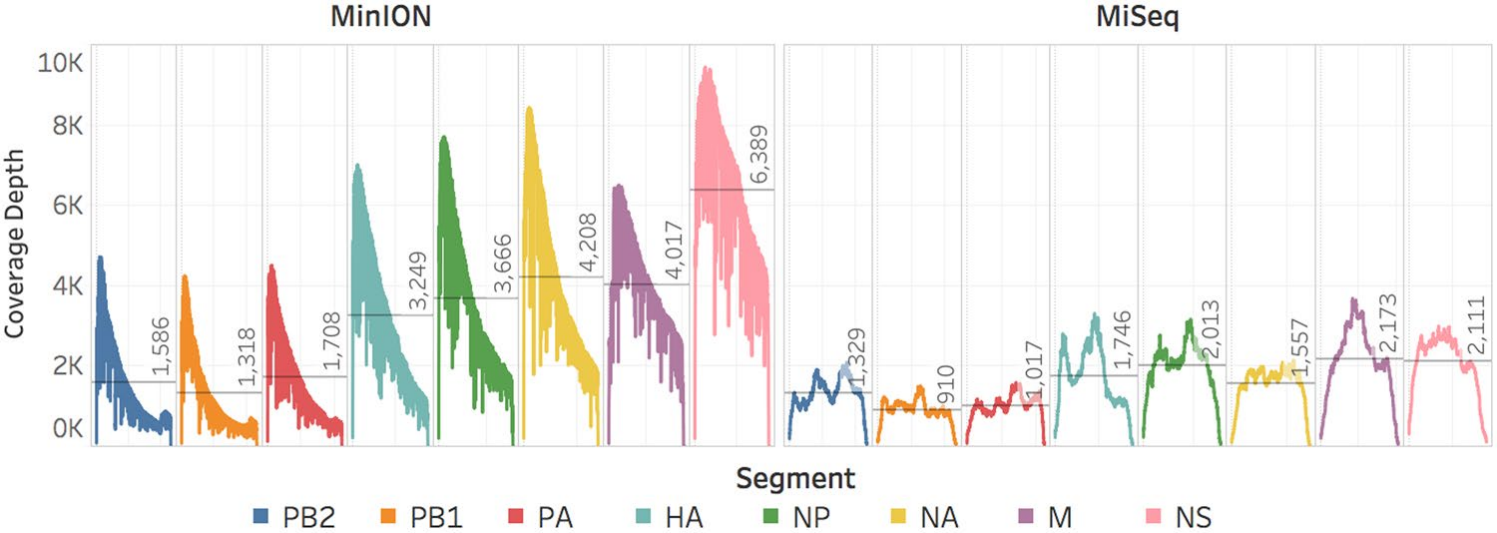
MinION direct RNA and MiSeq M-RTPCR sequencing covered the coding regions of the PB2, PB1, PA, HA, NP, NA, M, and NS genome segments of the influenza A virus genome from the influenza rA/Puerto Rico/8/1934 (H1N1) crude viral samples to an average depth of 2,789 and 1,478 respectively. Negative-sense slope coverages in the MinION results confirm the directionality of the sequencing and capture method.

**Figure 3:**
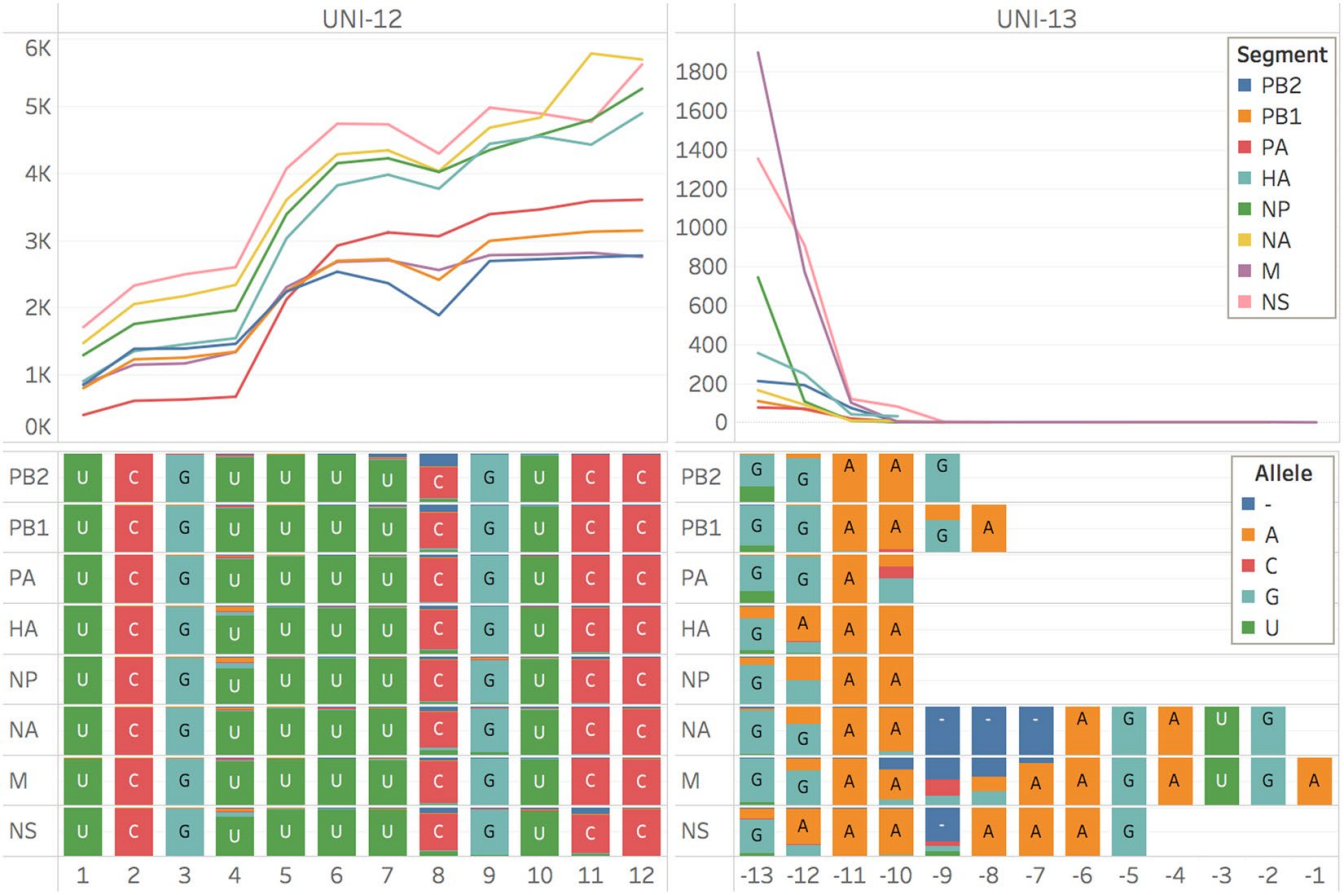
The extreme 3′ termini (Uni-12) of all segments were fully sequenced and matched the expected sequence with the exception of the degeneracy at the +4 position which was not resolved. The sequences for the extreme 5′ termini (Uni-13) that were obtained match the expected sequences with the exception of a C to G substitution at the −9 position in the segments PB1 and PB2. The loss of coverage at the extreme 5′ end of the molecule is most likely due to unreliable processivity as the last of the molecule passes and resulted in the final nine nucleotides not being sequenced in some of the segments. These missing bases in the extreme termini represent the difference between a coding complete genome, which is claimed here, and a complete genome^20^.

**Figure 4:**
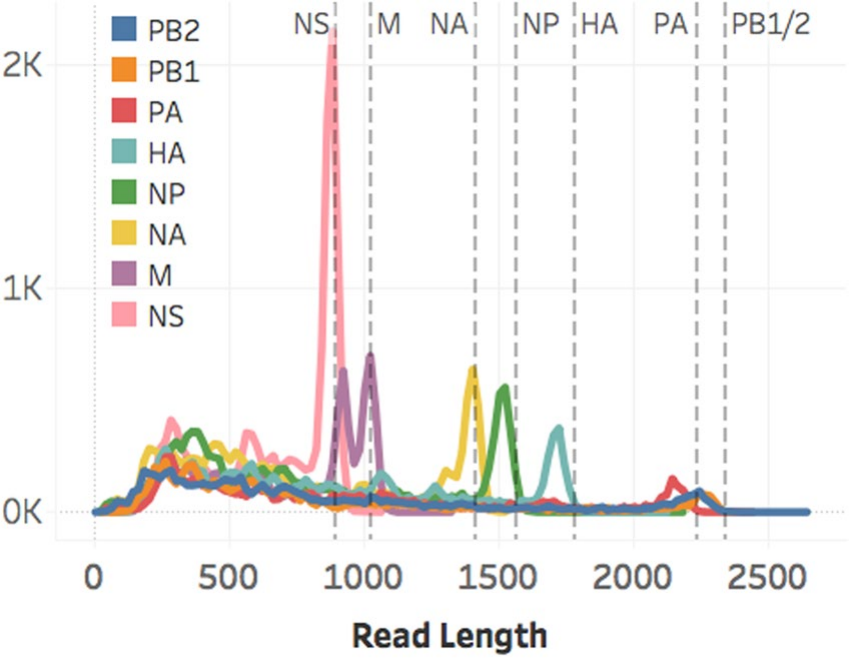
The aligned read length distributions correspond to the expected lengths (dashed lines) of the respective segments (NS 890 nt; M 1,027 nt; NA 1,413 nt; NP 1,565 nt; HA 1,778 nt; PA 2,233 nt; PB1 and PB2 2,341 nt) from the influenza rA/Puerto Rico/8/1934 (H1N1) crude viral samples. As the segment length increases, the read length distribution falls further short of the expected length, presumably due to RNA degradation. Aligned read lengths include insertion errors, accounting for the presence of reads larger than the expected value. Due to cases of large insertion errors, 14 total reads longer than 2,500 nucleotides were observed.

To determine the effectiveness of the modified adapter, total RNA from allantoic fluid (crude) harvested from a genetically defined recombinant virus (rA/Puerto Rico/8/1934 (H1N1)) infected chicken eggs (EID_50_ 6.8 × 10^9^) was sequenced via MinION. Three independent sequencing experiments each covered 100% of the coding regions of the PB2, PB1, PA, HA, NP, NA, M, and NS gene segments to an average depth of 2,789 ± 752 (Fig. 2) with reduced coverage at the extreme termini (Fig. 3). Since this approach reads from the 3′ to 5′ end of the molecule, there is a heavy coverage bias towards the 3′ terminus of the negative sense RNA. Of the 54,353 ± 15,314 reads, 98.8 ± 0.1% mapped to influenza rA/Puerto Rico/8/1934 (H1N1) virus (Tables 2 and S3) in a roughly even distribution among the 8 segments (Fig. S4), with 100% of the mapped reads in the negative-sense orientation. The distribution of read lengths (Fig. 4 and Table S1) corresponds well to the expected length of the respective segment. The read level accuracy was 86.2 ± 0.3%, and the consensus sequence was 98.97 ± 0.01% in concordance with consensus sequence generated using our standardized multi-segment reverse transcriptase polymerase chain reaction (M-RTPCR)^15,16^, Nextera, and MiSeq approach (Tables 2 and S3).

To compare the accuracy of the consensus sequence generated from direct RNA sequencing, the vRNA segments from the influenza rA/Puerto Rico/8/1934 (H1N1) pure and crude virus preparations were amplified by M-RTPCR^15,16^ and sequenced on the Illumina MiSeq. Sequencing of the RNA from purified virus and crude virus produced 163,264 and 143,572 reads, respectively, of which 99.9% mapped to influenza rA/Puerto Rico/8/1934 (H1N1) virus (Tables 2 and S3). The reads were roughly evenly distributed among the eight vRNA segments (Fig. S4). The mapped reads covered 100% of the coding regions of the PB2, PB1, PA, HA, NP, NA, M, and NS vRNA genome segments (Figs 2 and S3) with reduced coverage at the extreme termini (Fig. S6). The read level accuracy was 99.6% and the consensus sequences, which were used as the reference genome for the nanopore assemblies, were defined as 100% accurate and were 100% identical to each other.

### Contemporary influenza A viruses

To demonstrate that the adapter targets a region highly conserved among influenza A viruses, we directly sequenced vRNA from four contemporary influenza A viruses: A/Florida/20/2018 (H1N1pdm09), A/Texas/50/2012 (H3N2), A/chicken Ghana/20/2015 high pathogenic avian influenza (HPAI H5N1) and A/British Columbia/1/2015 low pathogenic avian influenza (LPAI H7N9) (Table 1). Single sequencing experiments demonstrated the coding complete genomic RNA was sequenced for each of the vRNA segments (PB2, PB1, PA, HA, NP, NA, M, and NS) with an average depth greater than 650 (Figs S7–S10 and Table S3). For these experiments, >96% of reads mapped to the respective influenza A virus genome generated by M-RTPCR and illumina MiSeq (Table 2). The high percentage of mapped reads from crude lysates indicates that the modified adapter effectively targets a diverse subset of influenza A viruses. All these contemporary influenza A viruses were sequenced via our standardized M-RTPCR^15,16^ amplified genome and MiSeq approach and that data was deposited in GenBank (NCBI).

### Limit of detection

The sensitivity of the direct RNA sequencing of influenza A virus strategy was determined through serial dilution of the RNA from influenza A/Florida/20/2018 (H1N1pdm09) virus. RNA was extracted and diluted fivefold serially to generate five RNA samples with Ct values: 11.6, 14.2, 17.0, 19.6, and 22.3 (Table S4). RNA was aliquoted and sequenced via MinION in triplicate. While some influenza A/Florida/20/2018 virus reads were detected in the dilute samples, the most dilute sample that yielded at least 10x coverage and 90% consensuses identity (Table S5) had a Ct of 17 and a calculated TCID_50_ of 1.89 × 10^7^. This is well outside the range of most original clinical samples and roughly four orders of magnitude less sensitive than M-RTPCR^15,16^.

## Discussion

We have demonstrated, for the first time, coding complete^20^ sequencing of an RNA virus genome by direct RNA sequencing. Using a method originally designed to sequence mRNA, we adapted the target sequence to bind the 3′ sequence conserved among influenza A viruses. The specificity of this adapter allowed efficient sequencing of influenza rA/Puerto Rico/8/1934 virus RNA genomic segments from RNA isolated from purified virus particles (control) or from RNA isolated from a crude extract that contains a myriad of viral and host (chicken) RNAs. Using this adapter, 98.8% of reads from the crude virus RNA preparation mapped to influenza rA/Puerto Rico/8/1934 virus, which is practically as efficient as with the purified virus RNA sample (99.3%). This performance on crude virus stocks demonstrates that the sequence-directed library preparation is a very effective method to select specific target RNA species among a population of RNAs, as the vast majority of reads were to influenza rA/Puerto Rico/8/1934 virus using 12 ribonucleotides as the target sequence.

The utility of this adapter was demonstrated by directly sequencing RNA from crude stocks of contemporary influenza A/Florida/20/2018 (H1N1pdm09), A/Texas/50/2012 (H3N2), A/chicken Ghana/20/2015 (H5N1), and A/British Columbia/1/2015 (H7N9) viruses. The adapter was able to target the conserved 3′ termini of this diverse subset of influenza A viruses as all four were sequenced to coding complete coverage and roughly 98% consensus identity to M-RTPCR and MiSeq results. Moreover, the adapter remained efficient with these diverse viruses with >96% of reads mapping to its respective influenza A virus genome.

The data shows that further modifications to the adapter could target other RNA species such as RNAs from specific pathogens and different RNA species within a particular pathogen. For example, one could compare (+) sense cRNA [replication intermediate of (−) sense vRNAs], (+) sense mRNAs, or (−) sense RNAs present during RNA virus infections (such as for influenza A viruses). The adapter sequence could be modified to target specific viral families, genera, or species by extending the target sequence and or by adding degeneracies. This is an advantage over poly(A) methods that have a reduced signal-to-noise ratio due to host mRNA. Targeting influenza A vRNA and cRNA independently may prove difficult as there is complementarity between the two conserved termini of the vRNA segments, and therefore high sequence identity between the 3′ termini of the (−) sense vRNA and (+) sense cRNA. Rather, cRNA and vRNA reads can be sorted based on their (+) and (−) polarity, respectively. Moreover, this technique is highly amenable to sequencing a variety of non-poly-adenylated RNAs from hosts and pathogens, including untranslated regions (UTRs), without biasing the sequence to the primer. This allows the examination of the UTRs in their native form, which we have done here with influenza A virus. Genomic length and quantitative sequencing of viral mRNA species, using unmodified kit components, has the potential to provide direct detection of base modifications, splice variants, and transcriptional changes. By examining (−) sense vRNA, native UTRs, (+) sense cRNA, viral mRNAs, and host mRNAs activated during an influenza infection, one could dissect the viral replication processes and observe changes at a given point in time and under different replication conditions, such as viruses used for vaccine production.

The primary limitations of this technology are the high read level error rate and high input material requirements. Reducing the error rate would enable multiplexing and more accurate consensus sequence determination and is a requirement for understanding nucleotide polymorphisms and genome sub-populations, particularly in viruses such as influenza that have significant intra-host diversity and or base modifications to be identified. There are currently several bioinformatic tools for detecting DNA base modifications such as Tombo, Nanopolish, SignalAlign, and mCaller; however, RNA specific tools have yet to be released^19^. Currently, the RNA input requirements for direct RNA sequencing are high and are not physically achievable with most original clinical samples. While we were able to successfully sequence influenza A vRNA using much less input material than is recommended by ONT, direct sequencing of serially diluted influenza A vRNA revealed that this technique is not sensitive enough for most clinical samples and roughly four orders of magnitude less sensitive than M-RTPCR based sequencing. Hence, direct RNA sequencing is currently limited to cultured viruses. Lessening the RNA input requirement of the direct RNA sequencing would take full advantage of the unbiased nature of direct RNA sequencing and allow for the detection and description of the rich diversity intrinsic to influenza and other viruses. The continuing effort to advance this technology by ONT will undoubtedly result in higher accuracy reads and greatly improved utility.

## Methods

### Concentration and purification of A/Puerto Rico/8/1934 reassortant virus

Genetically defined rA/Puerto Rico/8/1934 virus was created by reverse genetics^21^ and propagated in 11 day-old embryonated hen eggs at 35 °C for 48 hours. Allantoic fluid was harvested from the chilled eggs and clarified at 5,400 × g, 10 minutes, 4 °C (Sorvall SLA-1500 rotor). The virus was clarified twice more by centrifugation at 15,000 × g, 5 minutes, 4 °C (Sorvall SLA-1500 rotor). Virus was pelleted by centrifugation at 39,000 × g, 3 hours at 4 °C (Sorvall A621 rotor). Virus pellets were resuspended overnight in PBS and loaded onto a 30%/55% (w/w) density sucrose gradient. The gradient was centrifuged at 90,000 × g for 14 hours at 4 °C (Sorvall AH629 rotor). The virus fractions were harvested and sedimented at 131,000 × g (Sorvall AH629 rotor) for 2.5 hours. The resulting virus pellet was resuspended in PBS and aliquoted for future use.

### Propagation of contemporary influenza A viruses

A/Florida/20/2018 (H1N1pdm09), A/Texas/50/2012 (H3N2), and A/British Columbia/1/2015 were propagated in MDCK cells. A/chicken/Ghana/20/2015 was propagated in embryonated hen eggs and harvested as an E1/E3 passage.

### RNA isolation

Enolase II (YHR174W) mRNA is supplied in the ONT materials as the RNA calibration strand (RCS) at a concentration of 50 ng/µL. For influenza A virus samples, total RNA was isolated by Invitrogen^TM^ TRIzol® extraction^22^ according to manufacturer’s instructions with additional considerations for biosafety. The virus was inactivated by the addition of 10 volumes of TRIzol® in a Biosafely Level 2 biosafety cabinet. Influenza A/British Columbia/1/2015 (LPAI H7N9) and A/chicken Ghana/20/2015 (HPAI H5N1) viruses were inactivated by the addition of 3 volumes of TRIzol® in a Biosafely Level 3 enhanced laboratory before removal. Following inactivation, a fume hood was used for the chloroform addition and aqueous phase removal steps. RNA pellets were resuspended in 10–40 µL nuclease free water and quantified by Quant-iT^TM^ RiboGreen® RNA Assay Kit or a Qubit^TM^ RNA Assay Kit. Due to the difficulty in acquiring sucrose-purified material, the pure controls were limited to one MiSeq run and two separate MinION experiments. RNA from influenza A/Florida/20/2018 (H1N1pdm09) virus was diluted serially and aliquoted for determining the limit of detection.

### Nanopore Sequencing

The ONT direct RNA library preparation input material requirement is 500 ng of target molecule in a 9.5 µL volume (Table S6). For mRNA sequencing of the enolase control, the protocol was used according to the manufacturer’s instruction. For influenza vRNA sequencing, modifications were made to the protocol components (Table S2). We altered the supplied reverse transcriptase adapter (RTA) which has a T_10_ overhang (T_m_ ~ 20 °C) to target the ligation of the RTA to mRNA, with 12 nucleotides complementary to the conserved 3′ end of Influenza A vRNA^23^ (Fig. 1). RTA-U12 and RTA-U12.4 contained target sequences (5′ to 3′) AGC **A**AA AGC AGG and AGC **G**AA AGC AGG (T_m_ ~ 50 °C) respectively and were combined in a 2:3 molar ratio to a total concentration of 1.4 µM. This mixture was used as a direct replacement to the RTA supplied in the protocol for influenza A vRNA samples. Though there is some disagreement regarding the segment specific degeneracies of the 12 nucleotides at the 3′ end of the genome, RTA-U12 is expected to target the segments PA, NP, M, and NS; and RTA U-12.4 is expected to target the segments PB2, PB1, HA, and NA^24,25^. For the pure, crude, and contemporary virus experiments, 10 µL of vRNA was ligated to 1 µL of RTA-U12. For the LOD experiment, which also used influenza A (H1N1pdm09) virus, 9 µL of vRNA and 0.5 µL of 50 ng/µL enolase mRNA were combined and ligated to 1 µL of RTA-U12 and 1 µL of the stock RTA.

Adapter ligated RNA was directly sequenced on the MinION nanopore sequencing using a FLO-MIN107 flowcell equipped with the R9 chemistry. The enolase sequencing experiments were operated through MinKNOW versions 1.4.2, 1.7.7, and 1.10.11; the pure sequencing experiments were operated through MinKNOW 1.7.7; the crude, contemporary virus, and LOD 1 sequencing experiments were operated through MinKNOW 1.10.11; and the LOD 2–5 sequencing experiments were operated through MinKNOW 2.1. Raw data was basecalled using Albacore 2.1.10 (released 01/26/2018), and reads were assembled using IRMA^26^ with the FLU-MinION preset configuration to produce influenza A virus consensus sequences for comparison to MiSeq-derived consensuses. The FLU-MinION preset differs from the default FLU module settings by the following: dropping the median read Q-score filter from 30 to 0, raising the minimum read length from 125 to 150, raising the frequency threshold for insertion and deletion refinement from 0.25 to 0.75 and 0.6 to 0.75 respectively, and lowering the Smith-Waterman mismatch penalty from 5 to 3 and the gap open penalty from 10 to 6. For read-level comparisons of MinION to MiSeq, raw fastqs from both sequencing platforms were mapped with bwa-mem v.0.7.7 algorithm^27^ to MiSeq + IRMA derived consensus sequences as references. Bwa-mem settings were left default except for the following arguments: “-A 2” and “-B 3”. Figures and tables were created in Tableau v.10.4.3.

Error rates were calculated against the aligned plurality consensus sequence as follows:

- Accuracy rate = 1 − average number of insertions, deletions, and minority alleles/sum of aligned bases + number of deletions and insertions at left-adjacent (upstream or 5′ to the site) base per position per segment.
- Insertion rate = average number of insertions, irrespective of insertion length/sum of aligned bases + number of insertions at left-adjacent base per position per segment.
- Deletion rate = average number of deletions, irrespective of deletion length/sum of aligned bases + number of deletions at left-adjacent base per position per segment.
- Substitution rate = average number of minority bases/sum of aligned bases per position per segment.
- Alignment read lengths were calculated as matching + inserted bases per read (CIGAR M + I).

### Illumina MiSeq Sequencing

The coding complete influenza A virus genome was amplified with the RNA from all viral samples. The MRT-PCR used the Uni/Inf primer set^16^ with SuperScript III One-Step RT-PCR with Platinum Taq High Fidelity (Invitrogen). Following amplification, indexed paired-end libraries were generated from 2.5 µl of 0.2 ng/µL using the Nextera XT Sample Preparation Kit (Illumina) following the manufacturer protocol using half-volume tagmentation reactions. Libraries were purified with 0.8X AMPure XP beads (Beckman Coulter, Inc.) and assessed for fragment size (QIAxcel Advanced System, Qiagen) and quantitated using Quant-iT dsDNA High Sensitivity Assay (Invitrogen). Six pmol of pooled libraries were sequenced on the Illumina MiSeq with MiSeq v2 300 cycle kit and 5% PhiX spike-in to increase the sequence diversity. Sequence analysis was performed using IRMA^26^ as part of the current Illumina-based pipeline utilized by the Influenza Genomics Team at the Centers for Disease Control and Prevention.

## Electronic supplementary material

Supplementary Figures and Table Legends

Dataset 1

## Data Availability

Sequence data is accessioned at NCBI: PRJNA449380.
