# Author Correction: Direct RNA Sequencing of the Coding Complete Influenza A Virus Genome

**DOI:** 10.1038/s41598-018-34067-6

**Published:** 2018-10-19

**Authors:** Matthew W. Keller, Benjamin L. Rambo-Martin, Malania M. Wilson, Callie A. Ridenour, Samuel S. Shepard, Thomas J. Stark, Elizabeth B. Neuhaus, Vivien G. Dugan, David E. Wentworth, John R. Barnes

**Affiliations:** 1 Oak Ridge Institute of Science and Education (ORISE), Oak Ridge, Tennessee USA; 2 Battelle Memorial Institute, Atlanta, Georgia USA; 3 Influenza Division, National Center for Immunization and Respiratory Diseases (NCIRD), Centers for Disease Control and Prevention (CDC), Atlanta, Georgia USA

Correction to: *Scientific Reports* 10.1038/s41598-018-32615-8, published online 26 September 2018

In the original version of this Article, Matthew W. Keller and Benjamin L. Rambo-Martin were omitted as equally contributing authors.

The original version of this Article also contained typographical errors.

In the legend of Table 1 where:

“Influenza A/Florida/20/2018 (H1N1pdm09), A/Texas/50/2012 (H3N2), A/chicken Ghana/20/2017 (HPAI H5N1) and A/British Columbia/1/2015 (LPAI H7N9) viruses were used to demonstrate this method’s broad utility across contemporary influenza A viruses of current clinical significance”.

now reads:

“Influenza A/Florida/20/2018 (H1N1pdm09), A/Texas/50/2012 (H3N2), A/chicken Ghana/20/2015 (HPAI H5N1) and A/British Columbia/1/2015 (LPAI H7N9) viruses were used to demonstrate this method’s broad utility across contemporary influenza A viruses of current clinical significance”.

Additionally, in the Results section under subheading ‘Sequencing RNA from crude versus purified influenza rA/Puerto Rico/8/1934 (H1N1) virus’ where:

“The read level accuracy was 86.3 ± 0.3%, and the consensus sequence was 98.97 ± 0.01% in concordance with consensus sequence generated using our standardized multi-segment reverse transcriptase polymerase chain reaction (M-RTPCR)^15,16^, Nextera, and MiSeq approach (Tables 2 and S3)”.

now reads:

“The read level accuracy was 86.2 ± 0.3%, and the consensus sequence was 98.97 ± 0.01% in concordance with consensus sequence generated using our standardized multi-segment reverse transcriptase polymerase chain reaction (M-RTPCR)^15,16^, Nextera, and MiSeq approach (Tables 2 and S3)”.

These errors have now been corrected in the PDF and HTML versions of the paper.

