# Supplementary material for: Direct RNA Sequencing of the Coding Complete Influenza A Virus Genome
Source: Sci Rep. 2018 Sep 26;8:14408. doi: 10.1038/s41598-018-32615-8 (PMC6158192; doi:10.1038/s41598-018-32615-8)
Supplement: Supplementary file 1 — Supplementary Figures and Table Legends [file 41598_2018_32615_MOESM1_ESM.pdf]

Supplementary Information

Direct RNA Sequencing of the Coding Complete Influenza A Virus Genome

Matthew W. Keller[1], Benjamin L. Rambo-Martin[2], Malania M. Wilson[2], Callie A. Ridenour[2], Samuel S. Shepard[3], Thomas J. Stark[3], Elizabeth B. Neuhaus[3], Vivien G. Dugan[3], David E. Wentworth[3], and John R. Barnes[3]*

[1]Oak Ridge Institute of Science and Education (ORISE), Oak Ridge, Tennessee, USA
[2] Battelle Memorial Institute, Atlanta, Georgia, USA
[3]Influenza Division, National Center for Immunization and Respiratory Diseases (NCIRD), Centers for Disease Control and Prevention (CDC), Atlanta, Georgia, USA.

*Address correspondence to fzq9@cdc.gov

Figures

Figure S1

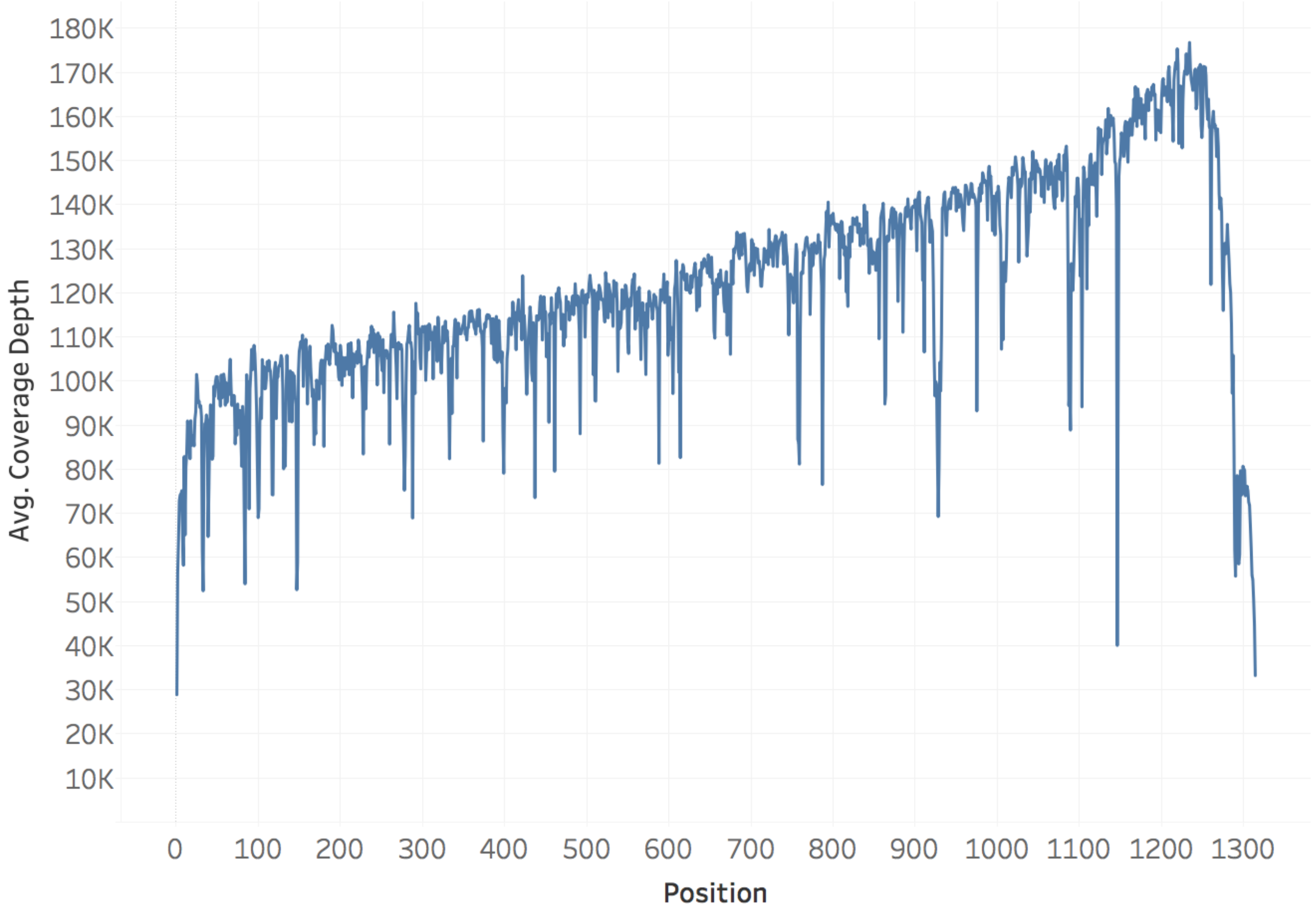

Average coverage of triplicate enolase direct RNA sequencing experiments. The MinION was able to sequence enolase mRNA to an average coverage depth of 122,207 ± 8,126 reads. The directional nature of nanopore sequencing results in a positive slope to the coverage for the mRNA.

Figure S2

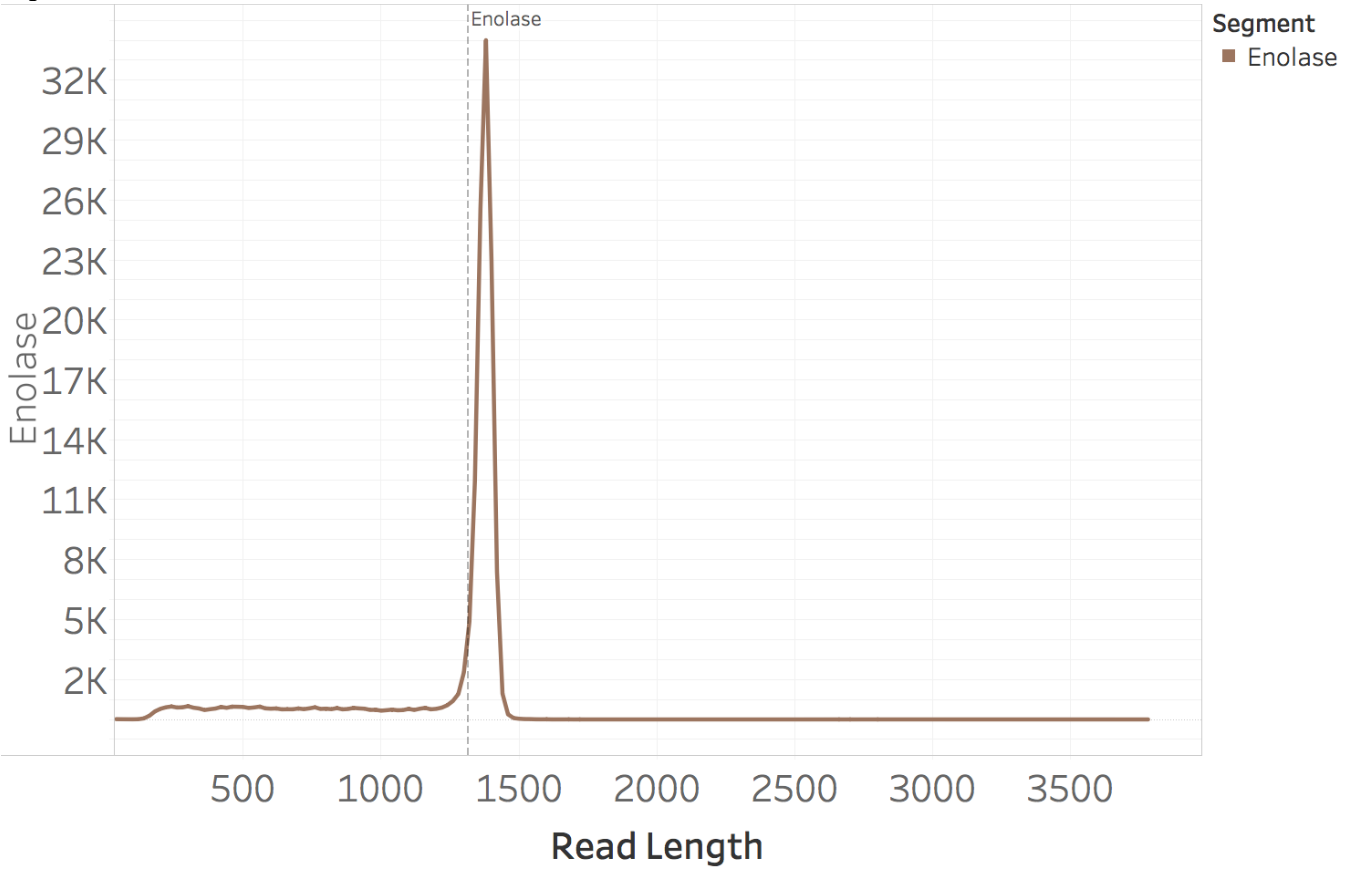

The aligned read length distribution is longer than the expected length of 1,314 nucleotides (dashed line) due to insertions.

Figure S3

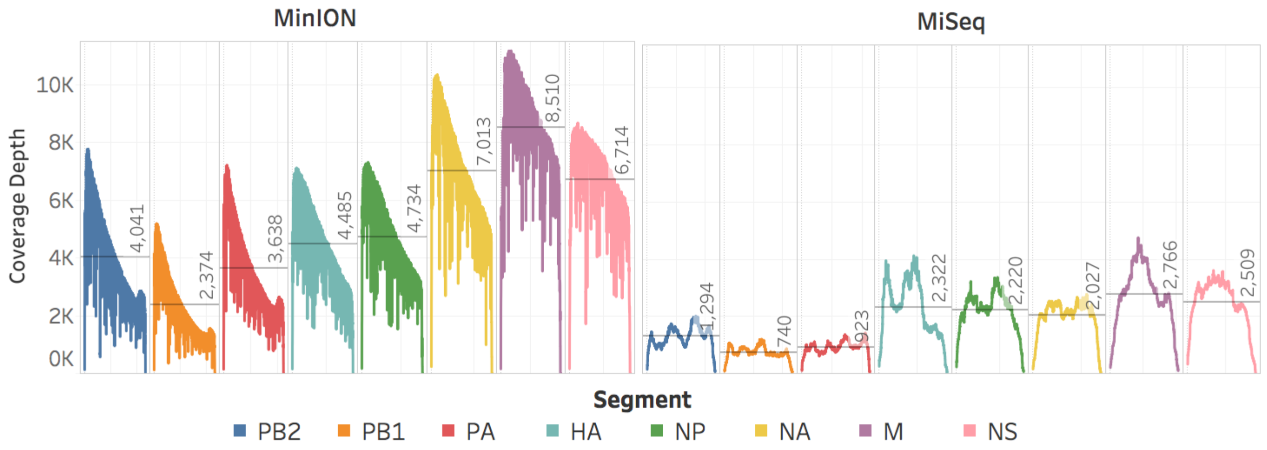

MinION direct RNA and MiSeq M-RTPCR sequencing covered the coding regions of the PB2, PB1, PA, HA, NP, NA, M, and NS genome segments of the influenza A virus genome from the influenza rA/Puerto Rico/8/1943 (H1N1) pure viral samples to an average depth of 4,648 and 1,646 respectively. Negative-sense slope coverages in the MinION results confirm the directionality of the sequencing and capture method.

Figure S4

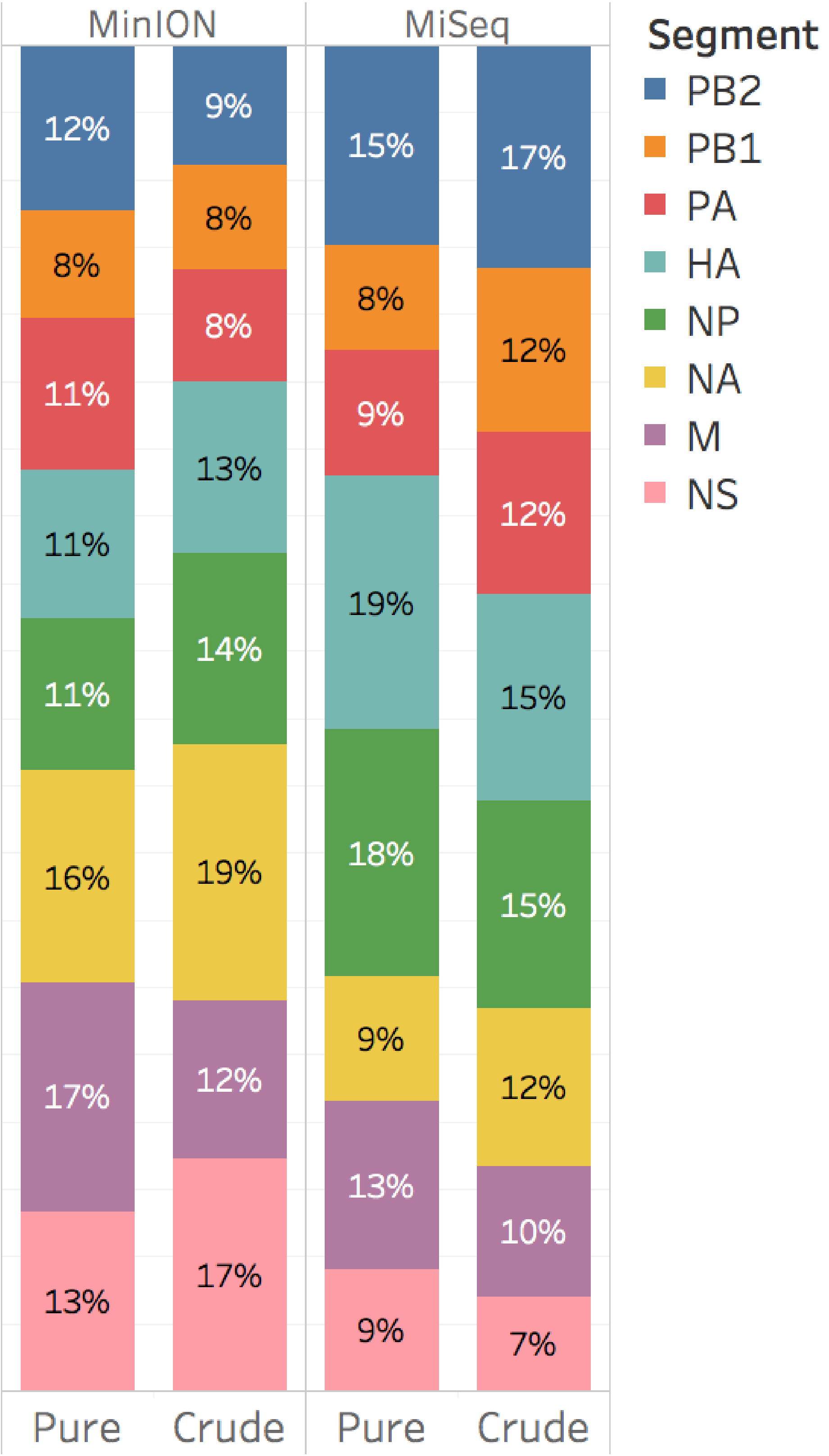

Distribution of mapped reads for all influenza rA/Puerto Rico/8/1942 (H1N1) virus experiment iterations.

Figure S5

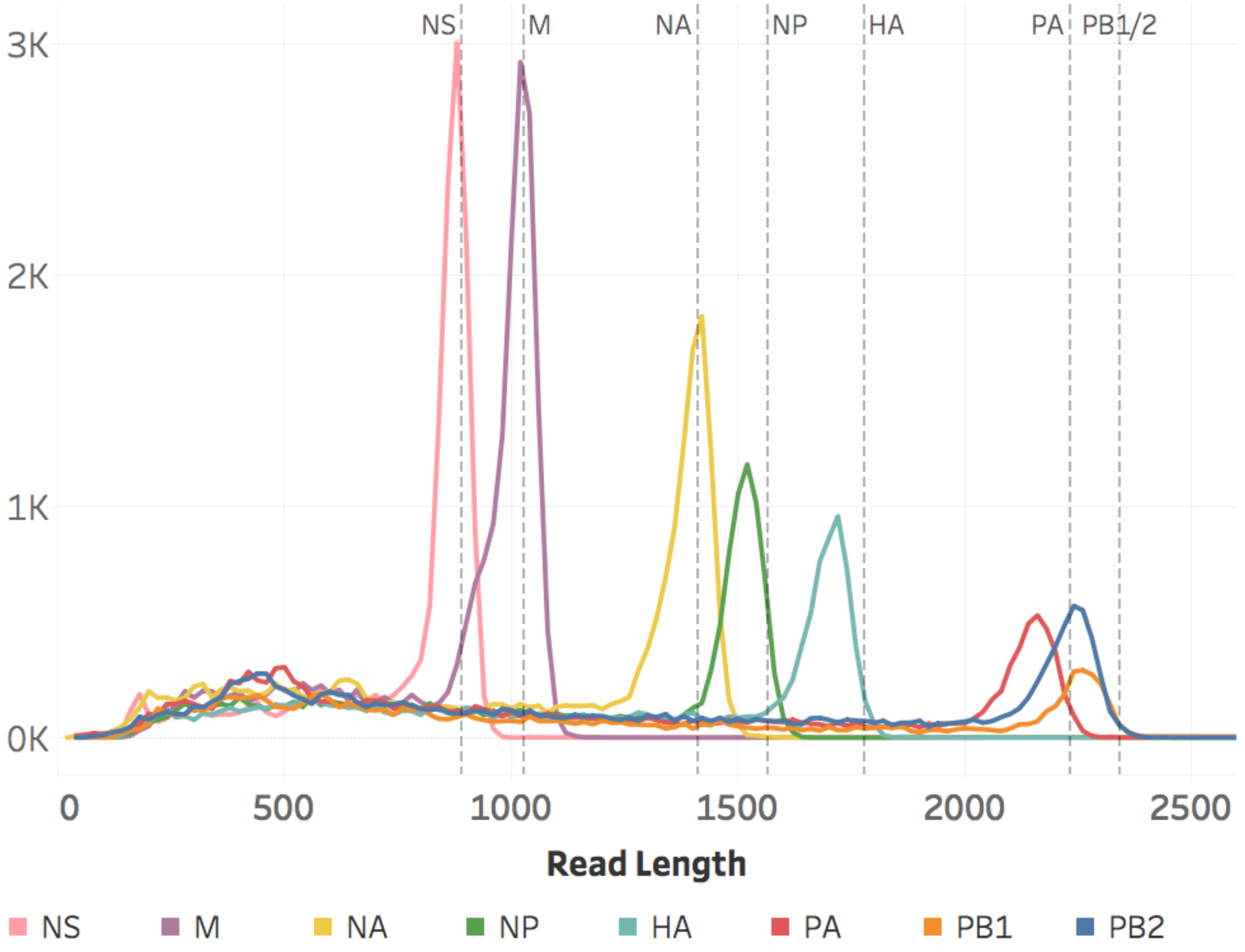

The aligned read length distributions correspond to the expected lengths (dashed lines) of the respective segments (NS 890 nt; M 1,027 nt; NA 1,413 nt; NP 1,565 nt; HA 1,778 nt; PA 2,233 nt; PB1 and PB2 2,341 nt) from the influenza rA/Puerto Rico/8/1934 (H1N1) pure viral samples. As the segment length increases, the read length distribution falls further short of the expected length, presumably due to RNA degradation. Aligned read lengths include insertion errors, accounting for the presence of reads larger than the expected value. Due to cases of large insertion errors, 14 total reads longer than 2,500 nucleotides were observed.

Figure S6

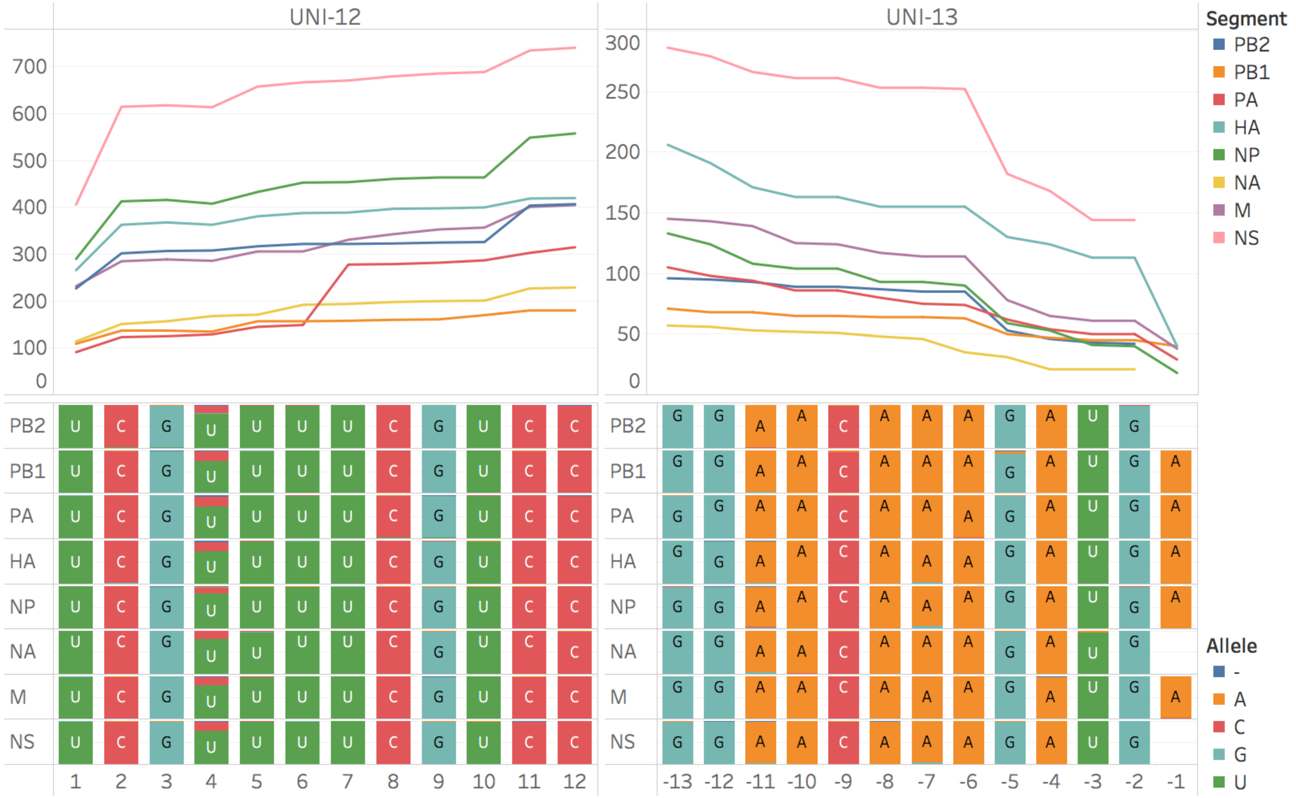

MiSeq coverage and consensus of the conserved termini of the influenza A viral genome segments from the influenza rA/Puerto Rico/8/1934 (H1N1) crude viral samples. These are the amplification sites, and the results are primer dictated sequences.

Figure S7

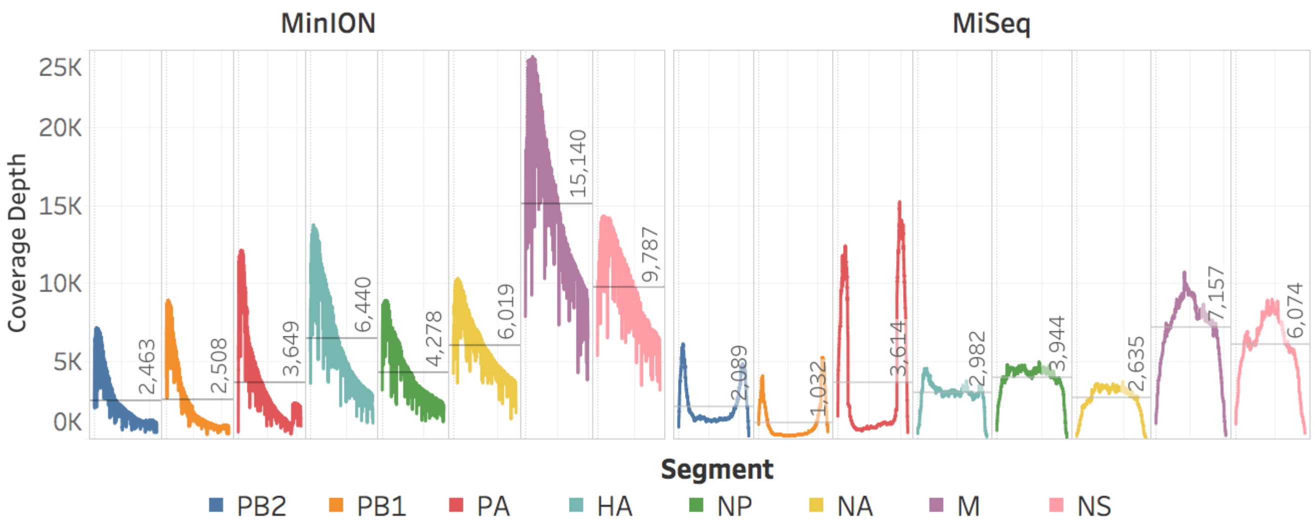

MinION direct RNA and MiSeq M-RTPCR sequencing covered the coding regions of the PB2, PB1, PA, HA, NP, NA, M, and NS genome segments of influenza A/Florida/20/2018 (H1N1pdm09) virus to an average depth of 5,203 and 3,189 respectively.

Figure S8

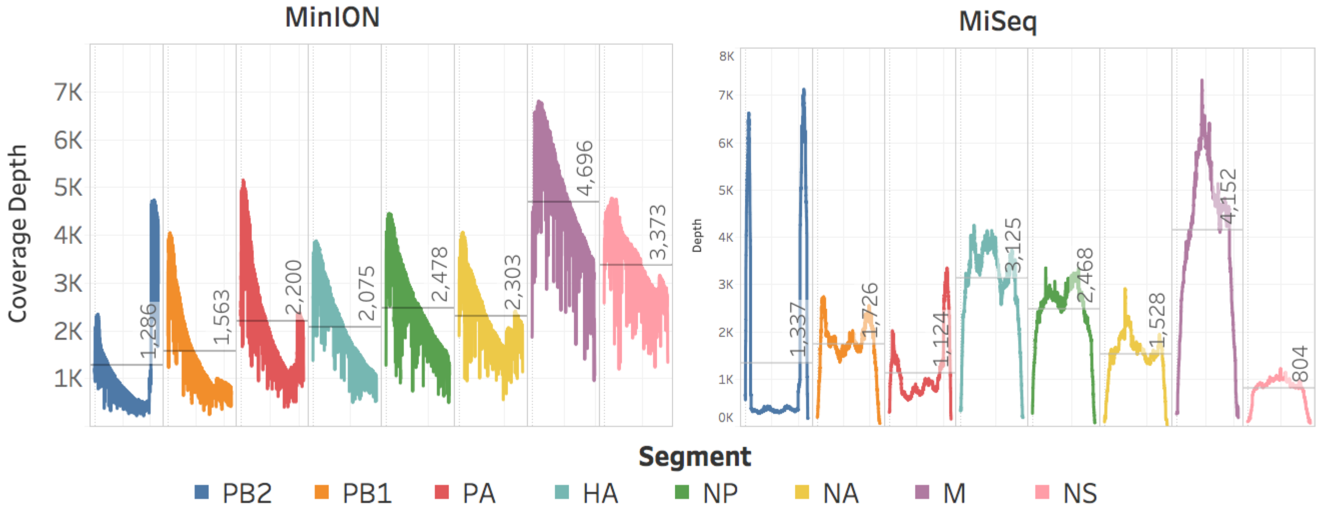

MinION direct RNA and MiSeq sequencing covered the coding regions of the PB2, PB1, PA, HA, NP, NA, M, and NS genome segments of influenza A/Texas/50/2012 (H3N2) virus to an average depth of 2,225 and 1,931 respectively.

Figure S9

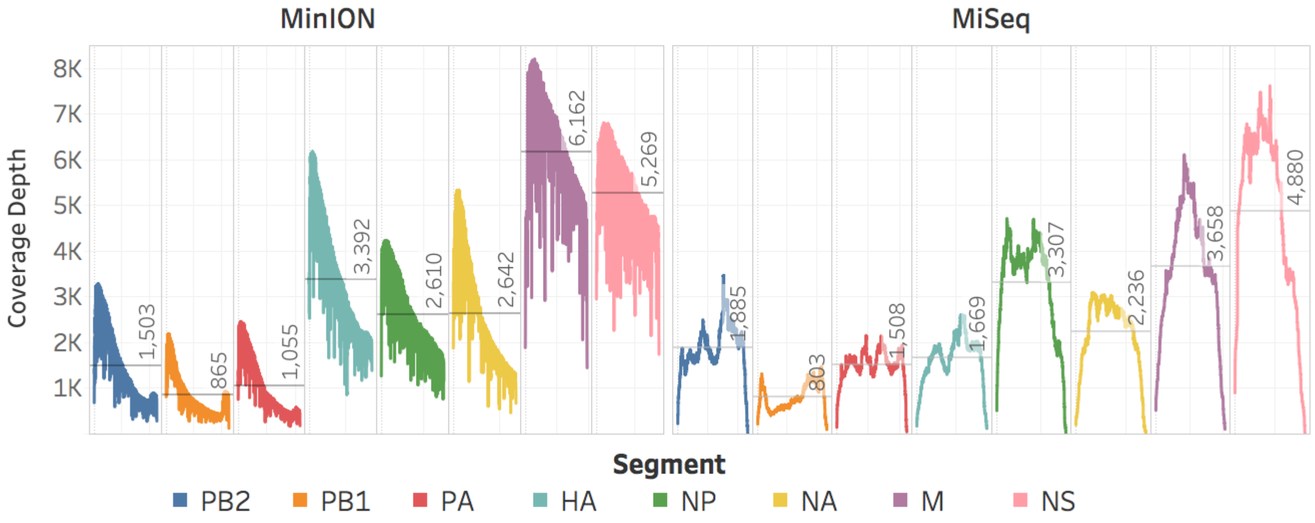

MinION direct RNA and MiSeq sequencing covered the coding regions of the PB2, PB1, PA, HA, NP, NA, M, and NS genome segments of influenza A/chicken Ghana/20/2015 high pathogenic avian influenza (HPAI H5N1) virus to an average depth of 2,411 and 2,139 respectively.

Figure S10

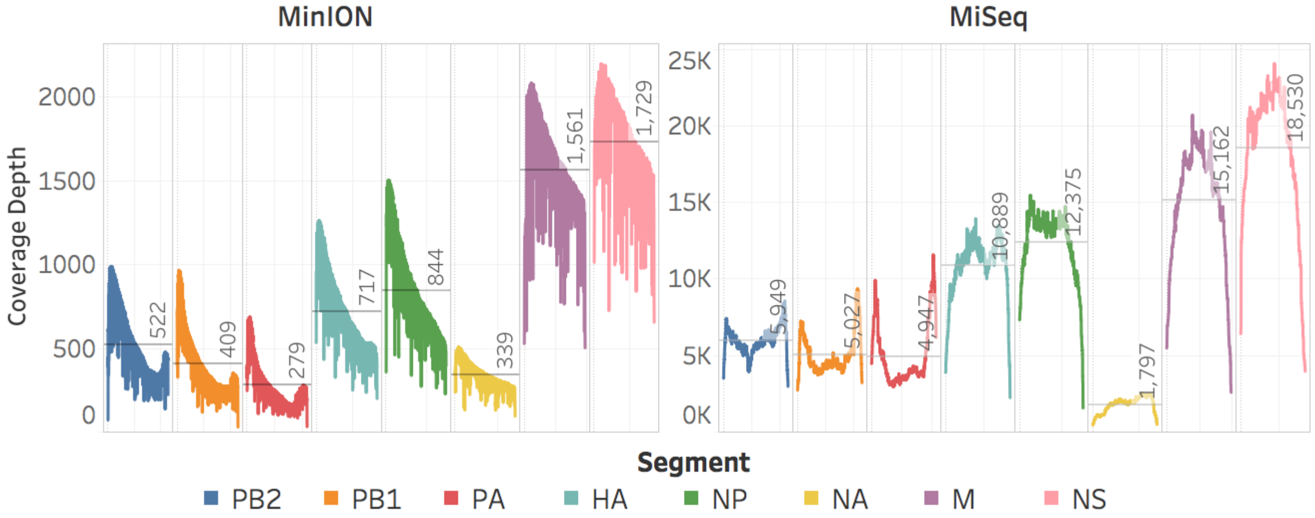

MinION direct RNA and MiSeq sequencing covered the coding regions of the PB2, PB1, PA, HA, NP, NA, M, and NS genome segments of influenza A/British Columbia/1/2015 low pathogenic avian influenza (LPAI H7N9) virus to an average depth of 652 and 8,101 respectively.

Table Legends

Table S1

Average and mode mapped read length is shown for MinION direct RNA sequencing experiments of RNA from pure and crude stocks of influenza rA/Puerto Rico/8/1934 (H1N1) virus. The presence of short reads, particularly in the crude sample, move the average read length much lower than the mode read length that is displayed here and in **figures 4, S2, and S5**. The read length distribution of the polymerases are all bimodal with an abundance of short reads along with full length reads. The read length distribution of M from the crude viral sample is also bimodal with a clear and well-defined peak at 920 nucleotides in addition to the full-length peak at 1,020 nucleotides. All Illumina reads were 150 nucleotides in length.

Table S2

Full sequences (5’ to 3’) of the adapters used in this study. Each RTA-B is duplexed with RTA-A. The stock RTA was supplied with the direct RNA sequencing materials. The modified RTAs were purchased from IDT with each of the modified RTA-B strands already duplexed to the RTA-A strand. The RTA-A has a 5’ phosphate modification for ligation. The regions of reverse complementarity between the RTA strands are underlined, and the target sequences are colored.

Table S3

Sequencing results are listed for: enolase via MinION in triplicate; purified influenza rA/Puerto Rico/8/1934 (H1N1) virus via MinION in duplicate and via MiSeq in singlicate; crude influenza rA/Puerto Rico/8/1934 (H1N1) virus via MinION in triplicate and MiSeq in singlicate; and influenza A/Florida/20/2018 (H1N1pdm09), A/Texas/50/2012 (H3N2), A/chicken Ghana/20/2015 (HPAI H5N1), and A/British Columbia/1/2015 (LPAI H7N9) viruses via MinION in singlicate.

Table S4

Fivefold serial dilutions of influenza A/Florida/20/2018 (H1N1pdm09) vRNA were used to determine the sensitivity of direct RNA sequencing. Ct values were directly measured from the RNA dilution. $TCID_{50}$ values were calculated from the original $TCID_{50}$ (3.16×10^7) and the Ct values of the original sample (9.6) and the dilutions. RNA was aliquoted and sequenced in triplicate (**Table S5**)

Table S5

Results of sequencing the LOD RNA dilutions (**Table S4**) are listed. Diluted vRNA was combined with 25 ng of enolase mRNA for library preparation and sequencing. The enolase signal is maintained while the influenza signal abruptly deteriorates.

Table S6

Input material and sequencing information for the MinION direct RNA sequencing experiments. The LOD input material does not include the addition of 25 ng of enolase mRNA though that RNA is present in the final library measurement. Pore occupancies for experiments in 2017 were estimated by ONT technical support from QC reports. Later runs were estimated directly during the experiment. Pore occupancy of the active channels is defined as channels in strand / (channels in strand + single pore channels) * 100%.

*Pore availability for mux 1-3 is displayed. These data were be recovered by ONT technical support for Enolase 1 and Pure 1. Mux 4 scan data was not able to be recovered for these samples. The mux 4 pores are fewer in number and are used lastly in long sequencing experiments or not at all in shorter experiments.
